# Análise da Lipoproteína de Baixa Densidade em Pacientes Diabéticos a Partir das Equações de Martin/Hopkins e Sampson

**DOI:** 10.36660/abc.20220859

**Published:** 2023-05-17

**Authors:** Cristian Rodrigues do Nascimento, Rodrigo Mendes, Carlos Alberto de Lima Botelho, Romero Henrique de Almeida Barbosa, Pedro Pereira Tenório

**Affiliations:** 1 Universidade Federal do Vale do São Francisco Paulo Afonso BA Brasil Universidade Federal do Vale do São Francisco, Paulo Afonso, BA – Brasil; 2 Irmandade Santa Casa de Misericórdia de São Paulo São Paulo SP Brasil Irmandade Santa Casa de Misericórdia de São Paulo, São Paulo, SP – Brasil

**Keywords:** Doenças Metabólicas, Dislipidemias, Doença Arterial coronariana, Diabetes Mellitus, Lipoproteína de Baixa Intensidade

Ao Editor,

Lemos com muito interesse o artigo: Comparação das Novas Equações de Martin/Hopkins e Sampson para o Cálculo do Colesterol de Lipoproteína de Baixa Densidade em Pacientes Diabéticos. O estudo comparou as equações de Martin/Hopkins (LDL-Cmh), de Sampson (LDL-Cs) e a de Friedewald (LDL-Cf) com a lipoproteína de baixa densidade obtida de forma direta (LDL-Cd). O intuito foi analisar qual delas teria maior concordância com LDL-Cd em pacientes diabéticos, além de investigar até que ponto essas novas equações poderiam mudar a tomada de decisão clínica em comparação com a LDL-Cf.^1^

Já está estabelecido na literatura que a lipoproteína de baixa densidade (LDL-c) está fortemente associada com doenças cardiovasculares (DCV), sendo que os pacientes com diabetes mellitus (DM) apresentam risco aumentado devido a capacidade aterogênica que as LDL-c promovem, sobretudo ao dano endotelial crônico desenvolvido pelo estado hiperinflamatório da hiperglicemia persistente.^2^ Logo, a medição precisa do LDL-c é fundamental para o seguimento clínico destes pacientes.

No estudo, o uso do LDL-Cmh e LDL-Cs apresentaram uma relação de forte concordância com LDL-Cd, não necessitando reclassificar os indivíduos. Ambas as equações se mostraram melhores do que a LDL-Cf, sobretudo os níveis de triglicerídeo (TG) >150 mg/dl. A LDL-Cmh teve uma concordância quase excelente com o LDL-Cd em indivíduos que portavam valores de TG entre 150-400 mg/dl, sendo necessário reclassificar apenas 1,5% dos pacientes quando usada a LDL-Cmh. Contudo, todas as equações tiveram um mal comportamento com a concentração de TG>400 mg/dl, possibilitando apenas que menos de 90% dos indivíduos fossem classificados corretamente. Além disso, a concordância entre LDL-Cd e LDL-c calculada foi ruim quando o valor do LDL-c foi < 70mg/dl.^1^

Os autores do estudo afirmam que as equações do LDL-Cmh e LDL-Cs apresentaram concordância muito similar ao LDL-Cd. Assim, a tomada de decisão clínica deve ser semelhante na maioria dos pacientes, independentemente de qual equação for usada. Para aqueles indivíduos com TG de 150 a 400 mg/dl, a LDL-Cmh apresentou uma concordância quase perfeita com o LDL-Cd, em relação a estar dentro da meta de LDL-C, fazendo com que ela seja preferível para portadores de DM nessa faixa de TG.^1^

Contudo, percebeu-se que no processo de obtenção da amostra populacional, mesmo se tratando de pacientes diabéticos, a análise estatística considerou apenas a variável TG sobre o comportamento de concordância, subestimação e superestimação nas equações analisadas. Logo, o DM por se tratar de um processo crônico, multifatorial, com diversas apresentações clínicas e abordagens terapêuticas, pode ter havido fatores de confusão na análise do comportamento das equações nos indivíduos diabéticos, pois, os pacientes não foram estratificados quanto ao tipo de DM, classe de antidiabéticos utilizados, adesão não-medicamentosa e medicamentosa, parâmetros antropométricos e função hepática.
