# Supplementary material for: Análise da Lipoproteína de Baixa Densidade em Pacientes Diabéticos a Partir das Equações de Martin/Hopkins e Sampson
Source: Arq Bras Cardiol. 2023 May 17;120(5):e20220859. [Article in Portuguese] doi: 10.36660/abc.20220859 (PMC10348386; doi:10.36660/abc.20220859)
Supplement: Translation

# Translation: Analysis of low-density lipoprotein in diabetic patients using the Martin/Hopkins and Sampson equations

**Authors:** Cristian Rodrigues do Nascimento, Rodrigo Mendes, Carlos Alberto de Lima Botelho, Romero Henrique de Almeida Barbosa, Pedro Pereira Tenório

**Affiliations:** 1 Brazil Universidade Federal do Vale do São Francisco, Paulo Afonso, BA – Brazil; 2 Brazil Irmandade Santa Casa de Misericórdia de São Paulo, São Paulo, SP – Brazil

**Keywords:** Metabolic diseases, Dyslipidemia, Coronary artery disease, Diabetes Mellitus, Lipoprotein LDL

Dear Editor,

We read with great interest the article: Comparison of Novel Martin/Hopkins and Sampson Equations for Calculation of Low-Density Lipoprotein Cholesterol in Diabetic Patients. The study compared the Martin/Hopkins (LDL-Cmh), Sampson (LDL-Cs), and Friedewald (LDL-Cf) equations with directly obtained low-density lipoprotein (LDL-Cd). The aim was to analyze which would have a greater agreement with LDL-Cd in diabetic patients and investigate how these new equations could change clinical decision-making compared to LDL-Cf.^1^

It is already established in the literature that low-density lipoprotein (LDL-c) is strongly associated with cardiovascular diseases (CVD), and patients with diabetes mellitus (DM) are at increased risk due to the atherogenic capacity that LDL-c promotes, mainly to chronic endothelial damage developed by the hyperinflammatory state of persistent hyperglycemia.^2^ Therefore, accurate measurement of LDL-c is essential for the clinical follow-up of these patients.

In the article, the use of LDL-Cmh and LDL-Cs showed a strong correlation with LDL-Cd, not requiring the reclassification of individuals. Both equations were better than LDL-Cf, especially triglyceride (TG) levels > 150 mg/dl. LDL-Cmh had an almost excellent agreement with LDL-Cd in individuals who had TG values between 150-400 mg/dl, requiring only 1.5% of patients to be reclassified when using LDL-Cmh. However, all equations behaved badly when the TG concentration was > 400 mg/dl, allowing only less than 90% of the individuals to be classified correctly. Furthermore, the concordance between LDL-Cd and calculated LDL-c was poor when the LDL-c value was < 70 mg/dl.^1^

The article’s authors state that the equations for LDL-Cmh and LDL-Cs showed similar concordance for LDL-Cd. Thus, clinical decision-making should be similar in most patients, regardless of which equation is used. For those individuals with a TG of 150 to 400 mg/dl, LDL-Cmh presented an almost perfect agreement with LDL-Cd, concerning being within the LDL-C target, making it preferable for patients with DM in this range of TG.^1^

However, it was noticed that in the process of obtaining the population sample, even in diabetic patients, the statistical analysis considered only the TG variable on the behavior of agreement, underestimation, and overestimation in the analyzed equations. Therefore, since DM is a chronic, multifactorial process with different clinical presentations and therapeutic approaches, there may have been confounding factors in analyzing the behavior of the equations in diabetic individuals, because the patients were not stratified according to the type of DM, class of antidiabetics used, non-medication and medication adherence, anthropometric parameters, and liver function.
