# Supplementary material for: Análise da Lipoproteína de Baixa Densidade em Pacientes Diabéticos a Partir das Equações de Martin/Hopkins e Sampson
Source: Arq Bras Cardiol. 2023 May 17;120(5):e20220859. [Article in Portuguese] doi: 10.36660/abc.20220859 (PMC10348386; doi:10.36660/abc.20220859)
Supplement: Related texts

# Reply: Carta-resposta

**Authors:** Abdulrahman Naser

A hipercolesterolemia é um determinante primário da aterosclerose e complicações da doença aterosclerótica em pacientes com diabetes. A identificação correta da concentração de lipoproteína de baixa densidade (LDL) no sangue é de extrema importância para determinar o risco de aterosclerose associada à hipercolesterolemia nesses pacientes. Devido à disponibilidade limitada de medição direta de LDL em centros de saúde, os laboratórios relatam as concentrações de LDL usando métodos de estimativa indireta. Desses métodos, a equação de Friedewald é o mais antigo e continua sendo o método mais comumente usado para estimar o LDL. Nosso estudo mostrou que novas equações foram superiores à antiga equação de Friedewald para avaliar se um paciente diabético estava dentro da meta de colesterol LDL recomendada pelas diretrizes. No entanto, a vantagem comparativa de usar qualquer equação sobre a fórmula de Friedewald foi mínima, e o risco associado à hipercolesterolemia pode ser classificado corretamente na maioria dos pacientes, independentemente da equação usada.^1,2^

Apesar de suas diferenças, as três equações usam as mesmas variáveis, ou seja, colesterol total, colesterol de lipoproteína de alta densidade (HDL) e triglicerídeos, para calcular o colesterol LDL. Dessas três variáveis, a concentração de colesterol total no sangue e a fração de colesterol transportada pelo colesterol de alta densidade são medidas diretamente e, portanto, não são responsáveis pelas variações observadas no cálculo indireto do colesterol LDL. O teor de colesterol da lipoproteína de densidade muito baixa (VLDL) é variável, e o teor de colesterol das partículas de VLDL é estimado usando as concentrações de triglicerídeos. A variação do teor de colesterol das partículas de VLDL é ainda mais pronunciada naqueles com triglicerídeos altos (ou seja, >400 mg/dl), e essa variação é responsável pela diferença nas estimativas de LDL quando diferentes equações são usadas. Estratificamos os pacientes em diferentes estratos de triglicerídeos para entender o desempenho das equações em pacientes com concentrações baixas ou altas de triglicerídeos, como feito em estudos semelhantes que analisaram o desempenho dessas equações em diferentes populações de pacientes.^3-5^

Embora as características individuais dos pacientes diabéticos, como o tipo e a gravidade do diabetes ou o número de medicamentos usados, sejam clinicamente importantes, os resultados atuais devem ser válidos independentemente de tais características individuais, uma vez que as mesmas concentrações sanguíneas de colesterol total, colesterol HDL, e triglicerídeos são usados em todas as três equações. Dito isso, o risco individual de mortalidade cardiovascular para um determinado paciente é, sem dúvida, afetado por tais características clínicas individuais. No entanto, infelizmente, nenhuma das ferramentas de estratificação de risco disponíveis considera todas as variáveis associadas ao risco de mortalidade cardiovascular em pacientes com diabetes. Embora esta última preocupação seja uma fonte potencial de erros, tais erros decorrem da relativa imperfeição das ferramentas de estratificação de risco disponíveis e não estão diretamente relacionados à nossa metodologia de análise.

# Reply

**Authors:** Abdulrahman Naser

Hypercholesterolemia is a primary determinant of atherosclerosis and complications of atherosclerotic disease in patients with diabetes. Correct identification of blood low-density lipoprotein (LDL) concentration is of utmost value to determine the risk of atherosclerosis associated with hypercholesterolemia in such patients. Due to the limited availability of direct measurement of LDL in healthcare centers, laboratories report LDL concentrations using indirect estimation methods. Of those methods, the Friedewald equation is the oldest and remains the most commonly used method to estimate LDL. Our study showed that novel equations were superior to the older Friedewald equation in assessing whether a diabetic patient was within the guideline-recommended LDL cholesterol target. However, the comparative advantage of using either equation over the Friedewald formula was rather minimal, and the risk associated with hypercholesterolemia could be correctly classified in most patients regardless of the equation used.^1,2^

Despite their differences, all three equations use the same variables, namely total cholesterol, high-density lipoprotein (HDL) cholesterol, and triglycerides, to calculate LDL cholesterol. Of those three variables, the total blood cholesterol concentration and the fraction of cholesterol transported by high-density cholesterol are measured directly, and therefore they are not responsible for the variations seen in the indirect calculation of LDL cholesterol. The cholesterol content of very-low-density lipoprotein (VLDL) is variable, and the cholesterol content of VLDL particles is estimated using triglyceride concentrations. The variation of the cholesterol content of VLDL particles is even more pronounced in those with high (i.e.,>400 mg/dl) triglycerides, and this variation is responsible for the difference in LDL estimates when different equations are used. We have stratified the patients within different triglyceride strata to understand the performance of equations in patients with low or high triglyceride concentrations, as done in similar studies that analyzed the performance of these equations in different patient populations.^3-5^

While the individual characteristics of diabetic patients, such as the type and severity of diabetes or the number of drugs used, are clinically important, present results should nonetheless be valid regardless of such individual characteristics given that the same blood concentrations of total cholesterol, HDL cholesterol, and triglycerides are used in all three equations. That said, the individual risk of cardiovascular mortality for a given patient is undoubtedly affected by such individual clinical characteristics. However, unfortunately, none of the available risk stratification tools account for all variables associated with cardiovascular mortality risk in patients with diabetes. While this latter concern is a potential source of error, such errors stem from the relative imperfection of available risk stratification tools and are not directly related to our analysis methodology.
